# Simplified Tai Chi Program Training versus Traditional Tai Chi on the Functional Movement Screening in Older Adults

**DOI:** 10.1155/2016/5867810

**Published:** 2016-11-13

**Authors:** Huiru Wang, Ankui Wei, Yingzhi Lu, Bo Yu, Wenhua Chen, Yang Lu, Yang Liu, Dinghai Yu, Liye Zou

**Affiliations:** ^1^Department of Sport and Physical Education, Shanghai Jiao Tong University, Shanghai, China; ^2^School of Kinesiology, Shanghai University of Sport, Shanghai, China; ^3^Department of Rehabilitation Medicine, Shanghai First People's Hospital, Shanghai Jiao Tong University, Shanghai, China; ^4^School of Physical Education and Sport Training, Shanghai University of Sport, Shanghai, China; ^5^School of Chinese Wushu, Shanghai University of Sport, Shanghai, China; ^6^Department of Health Education and Physical Education, Springfield College, Springfield, MA 01109, USA

## Abstract

*Background*. The present study aimed to evaluate and compare the effect of two different types of Tai Chi programs on the Functional Movement Screening (FMS) in older adults.* Methods*. Ninety older adults (65.5 ± 4.6 years old) who met the eligibility criteria were randomized into three different groups based on a ratio of 1 : 1 : 1: a traditional Tai Chi exercise (TTC), a simplified Tai Chi exercise (TCRT), or a control group (routine activity). The FMS consisted of the deep squat, hurdle step, in-line lunge, shoulder mobility, active straight leg rise, trunk stability push-up, and rotatory stability, which was used to measure physical function before the present study and after six months of Tai Chi interventions.* Results*. Seventy-nine participants completed the present study (control = 27, TTC = 23, and TCRT = 29). Significant improvement on the FMS tests between the baseline and after the six-month intervention was observed in both Tai Chi programs, whereas no significant improvement was observed in the control group. In addition, participants in the TCRT group demonstrated greater improvement than those in the TTC group.* Conclusions*. The TCRT is more effective in improving the physical function in older adults when compared to the traditional Tai Chi modality, particularly for improving balance.

## 1. Background

Falls are one of the most devastating problems that emerge in more than 30% of older adults, leading to high morbidity and death rates. In particular, not only does fall-related morbidity significantly reduce the quality of life in older adults, but also it challenges the national healthcare system [1]. Potential risk factors have been identified in association with falls, including weak lower extremity, unstable gait, and limited mobility [2]. Researchers emphasize that older adults should pay attention to improving their muscular strength and mobility to help stabilize the body (balance), ultimately reducing rates of falling [3].

In recent years, exercise has been recommended as one of the most effective and cost-effective methods for fall prevention in older adults [4]. Traditional Chinese Qigong exercises (TCQG) (e.g., Taichi, Baduanjin, and Daoyishu), characterized by gracefulness, softness, mindfulness, and gentleness, were proven to have a positive impact on flexibility and functional balance contributing to fall reduction in older adults [5]. Two systematic reviews consistently support the effectiveness of Tai Chi on reducing the frequency of falls and fear of falling [6, 7]. For instance, a research group designed a randomized controlled study and found that a six-month Tai Chi intervention protocol positively affected the total number of falls and psychological fear of falling in older adults (aged 70 or above) with sedentary lifestyle [8].

Although people of different ages and health status received benefits from practicing Tai Chi, the complexity of moves of traditional Tai Chi has become a big challenge to Tai Chi practitioners, especially for those novice older adults. More specifically, the complex and lengthy traditional Tai Chi form does not simply challenge motor memory capability in older adults that could discourage them, but relatively large space is demanded for home-based practice. A simplified Tai Chi form is therefore needed for older adults to strengthen their balance for fall prevention.

We therefore developed a simplified Tai Chi resistance training protocol consisting of 32 moves, which focus on improving physical function (e.g., balance, flexibility, and agility) and circulating flow of internal energy. Prior to the present study, the researchers designed a randomized controlled study to investigate the effectiveness of this simplified Tai Chi form in alleviating bone loss in menopausal women and found that the simplified Tai Chi form is as effective as the traditional Tai Chi form in slowing bone loss [9]. Whether this simplified Tai Chi could improve balance in older adults still remains unclear; therefore, the present study aimed to compare the effectiveness of the 8-minute Tai Chi form versus the traditional Tai Chi form on the Functional Movement Screen (FMS) in older adults when compared to a control group [10].

## 2. Materials and Methods

### 2.1. Participants

Participants in the present study were recruited from Shanghai City of China, through advertisements placed in local newspapers and in community centers. The demographic information of participants at the baseline is presented in Table 1. The study was approved by the Scientific Research Ethics Committee of Shanghai Sport University. Participants were included in the present study if they met the following eligibility criteria: (1) they were aged between 60 and 70 years; (2) they were employed in jobs without substantial physical demands; (3) they were currently not participating in any other supervised exercise program or did not attend any exercise program for the last three months; (4) they did not have any major diseases and/or physical illnesses (as measured by Manual Muscle Test) [11] that limit their practice of Tai Chi exercise; (5) they were not involved with any mental disorders (as measured by the Mini-Mental State Examination) [12] that may negatively affect understanding Tai Chi moves.

Medical history and health status were checked by a medical doctor from the Department of Rehabilitation, Shanghai First People's Hospital. The Manual Muscle Test for muscular strength, Goniometer for range of motion on both hips and knees, and the Mini-Mental State Examination for cognitive function were administered by another two medical doctors from the same hospital. All abovementioned screening took place prior to the beginning of randomized assignment. All eligible participants signed consent forms prior to being randomly placed into three groups (TTC = 30, TCRT = 30, and control = 30) based on computer-generated numbers.

### 2.2. Exercise Interventions

Four Tai Chi instructors (males = 2 and females = 2) were recruited from Shanghai Sport University. Taking into account the performance bias (standardized instruction), prior to the present study, the four instructors received three months of official training together, given by a Tai Chi grandmaster who was familiar with both TTC and TCRT. After the three months of the training, all instructors reached the standard of the effectiveness of instruction and then were equally assigned into the two Tai Chi groups based on mixed gender.

Participants in the control group were asked to keep their original lifestyles. Participants in the traditional Tai Chi group experienced four 60-minute Yang Style Tai Chi (85 moves) sessions weekly for six months. Of the four sessions weekly, two sessions were conducted by two experienced Tai Chi instructors and home-based practice for the remaining two sessions took place through watching a Tai Chi video. Participants in the TCRT had a similar protocol, involving frequency and duration of simplified Tai Chi program, and exercise modes (supervised and home-based). The exercise routine included a 10-minute warm-up, subsequent 40-minute Tai Chi form, and 10-minute relaxation at the end. The four instructors took attendance and reported it to researchers. Participants in the TCC group spent their first months learning and refining their moves before they practiced the entire routine. When compared to the 85-move TTC, participants in the TCRT group only spent their first two weeks in their learning stage because it included only 32 moves. The similarities and differences between simplified Tai Chi and traditional Tai Chi are presented in Table 2.

### 2.3. Functional Movement Measurement

Physical function was measured using the FMS consisting of seven tasks before and after the six-month Tai Chi intervention period. The seven tasks included deep squat (assessing bilateral, symmetrical, and functional mobility of the hips, knees, and ankles), hurdle step (assessing the bilateral functional mobility and stability of the hips, knees, and ankles), in-line lunge (assessing torso, shoulder, hip, and ankle mobility and stability, quadriceps flexibility, and knee stability), shoulder mobility (assessing bilateral shoulder range of motion, combining internal rotation with adduction and external rotation with abduction), active straight leg rise (assessing active hamstring and gastric-soleus flexibility while maintaining stable pelvis and active extension of the opposite leg), trunk stability push-up (assessing trunk stability in the sagittal plane while a symmetrical upper extremity motion is performed), and rotary stability (assessing multiplane trunk stability during a combined upper and lower extremity motion) [10–13]. Two high-resolution cameras located in the sagittal and frontal planes (Sony HXR-NX3, China) were used to record the quality of movement while performing the FMS tasks. All tests took place in the indoor sport hall of Shanghai University of Sport.

Each task has specific score criteria for the rater to determine among 0, 1, 2, and 3. Any pain reported while performing the movement in each test automatically resulted in a score of 0. The participant received a score of 3 if the performance meets all the standards delineated in the manual. Each test was scored from 0 to 3, and the maximum total score is 21. The secondary author who is a certified FMS expert recruited two graduate students and gave them official training before the present study. In order to avoid detection bias, the two assessors were blinded to the purpose of the present study. First of all, the two assessors independently evaluated the quality of movement of each task. If any disagreement existed between the two assessors, the certified FMS expert intervened to resolve the disagreement. The interrater reliability for assessing each task of the FMS was 92% or above.

### 2.4. Statistical Analysis

A statistician who was blinded to the outcome measures performed data analysis. For the total score, an intention-to-treat analysis was performed to compare among the three groups. The effects of the interventions were assessed by using analysis of covariance (ANOVA) for repeated measures within 3 (group: TCRT group, TTC group, and control group) × 2 (time: before and after). The effect was localized by using LSD's correction for multiple comparisons. Other than the repeated measure, the percentage differences (0–6 months) were calculated from duration between baseline and the end of the measurements for each individual. Furthermore, for each individual FMS test, crosstab was used to calculate the population percentage of improving, remaining, and descent. All analyses were done using SPSS version 20, and *p* < 0.05 was considered a statistically significant difference.

## 3. Results

**Figure 1:**
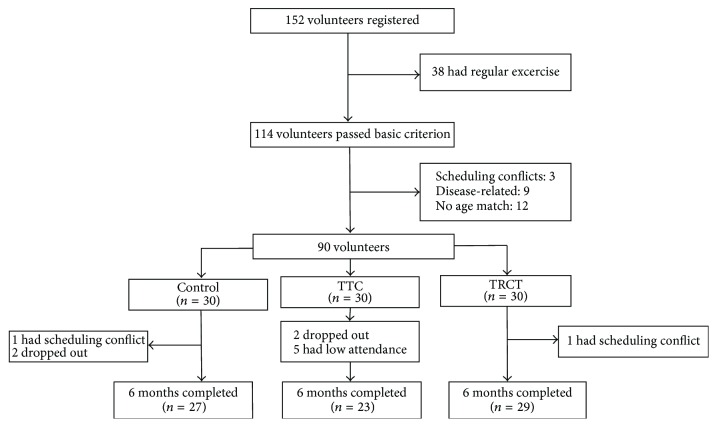
The entire process of screening, randomization, and completion of the 6-month intervention.

The research group had received responses from 114 participants who were interested in participating in the present study. Based on the eligibility criteria, 24 volunteers were excluded (age = 12, disease = 9, and schedule conflict = 3). A final number of 90 participants (65.5 ± 4.6 years) were included in the present study. Seventy-nine participants completed the six-month Tai Chi intervention period: 3 (schedule conflict = 1 and dropout = 2) in the control group, 7 (dropout = 2 and low attendance = 5) in the TTC, and 1 with schedule conflict in the TCRT group.  Figure 1 presents screening, randomization, and completion of the 6-month intervention.

**Figure 2:**
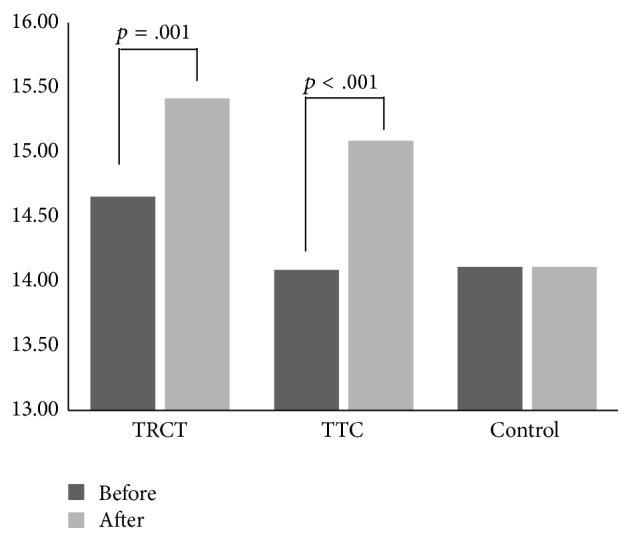
The interaction between the time and groups.

For the total score, the main effect of time was significant (*F*(1, 76) = 17.726; *p* < .001; *η*_*p*_^2^ = .189), and a significant improvement in both Tai Chi groups was observed before and after the six-month Tai Chi intervention period (14.284 ± 1.417 versus 14.871 ± 1.522; *p* < .001). The main effect of group was also significant (*F*(2, 76) = 3.640; *p* = .031; *η*_*p*_^2^ = .087), and pairwise comparisons showed that TCRT had higher scores than the control group (15.034 ± 1.395 versus 14.111 ± 1.489; *p* = .009). Finally, the interaction between time and group was found (*F*(2, 76) = 3.514; *p* = .013; *η*_*p*_^2^ = .109), with further analysis showing that both the TCRT (14.655 ± 1.289 versus 15.414 ± 1.500; *p* = .001) and the TTC (14.087 ± 1.443 versus 15.087 ± 1.276; *p* < .001) groups had improved total score, while no difference was found in the control group (14.111 ± 1.502 versus 14.111 ± 1.476; *p* = 1.000) (Figure 2).

Table 3 presents the percentage of improvement, maintenance, and declining on each task of the FMS across three groups.

**Figure 3:**
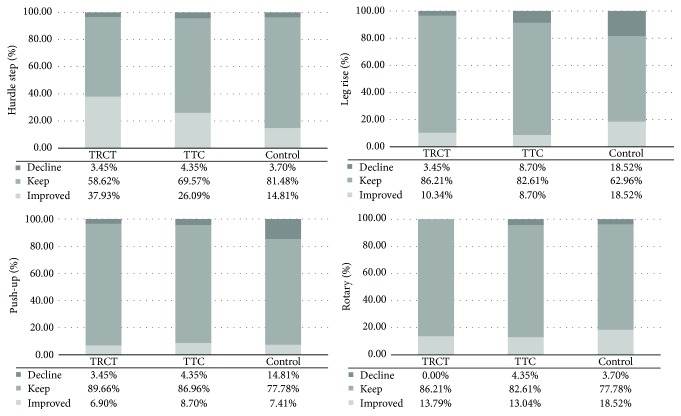
The percentage of change for the four tasks of the FMS across three groups.

With respect to each individual task of the FMS, the percentage of change in the TCRT group was observed in hurdle step (improvement: 37.93%, decline: 4.35%), leg rise (improvement: 10.34%, decline: 4.35%), push-up (improvement: 6.90%, decline: 4.35%), and rotary (improvement: 13.79%, decline: 0.00%) test with the least decline percentage and relative high improvement percentage, compared with the TTC and control group (Figure 3). The change of percentage at the baseline and after the six-month Tai Chi intervention period is reported in Table 3.

## 4. Discussion

The present study was to evaluate and compare the effect of two different types of Tai Chi training programs on the Functional Movement Screening (FMS) in older adults: a simplified Tai Chi resistance training program and traditional Tai Chi. Older adults who experienced either Tai Chi program experienced significant improvement on balance performance after the six-month intervention period.

Although many factors may contribute to falls, it has been documented that weak muscular strength in the lower extremities and balance ability due to aging are the main risk factors. Physical exercise has been proven to improve muscular strength and balance ability for fall prevention in older adults [14, 15]. Tai Chi is a safe, health-enhancing exercise and is suitable for older adults due to the features of gentleness and softness [16]. Tai Chi has been extensively studied and its positive impact includes improving postural stability and alleviating degeneration of muscular strength in older adults [17]. Shih [18] found that, after 16 weeks of Tai Chi training, the dynamic sway velocity as an indicator of balance became significantly lower among older people. After a 16-week Tai Chi practice, researchers found that older adults demonstrated significant improvement on the postural stability test due to decreasing displacement of center of pressure under the feet [19].

Tai Chi practice is effective in strengthening the limb muscle groups that contribute to initiating voluntary, goal-directed, and coordinative movements for postural stability [20]. The traditional Tai Chi routine is lengthy and complex, which restricts those people who lack the time, commitment, or mobility to exercise in groups under the supervision of an instructor. The simplified Tai Chi exercise not only keeps the original essence of the traditional Tai Chi improving physical function, but also is more easily accessible to older adults [16]. The resistance training in the simplified Tai Chi exercise has been proven to have a positive impact on alleviating bone mass loss [16]. The present study also showed that the simplified Tai Chi improved the FMS scores, which may be attributed to its main function improving physical function. Among the seven FMS functional tests, the hurdle step and leg rise tests are two main indictors of physical balance. In the present study, we found that the simplified Tai Chi exercise had a greater impact on the hurdle step and leg rise than the traditional Tai Chi routine. The leg rise test reflects the flexibility of the posterior femoral muscle group, as well as the strength of quadriceps femurs, the condition of lumbar vertebrae, and the stability of the pelvis. The muscle-stretching exercise, the second series of the new Tai Chi exercise, is specialized in improving flexibility. The hurdle step test is used to evaluate the ability of lifting up the lower limb and striding over the obstacle (hurdle) while stabilizing the torso. It reflects the static stability of the torso, stability of the supporting leg, and the agility of the striding leg. Accidental falls in older adults are mainly attributed to weak lower extremity (e.g., single leg and double leg support and the difficulty to initiate a voluntary and coordinated movement due to nerve degeneration).

The present study supports the notion that the simplified Tai Chi exercise is as effective as the traditional Tai Chi routine in improving physical function, particularly for balance. When compared to the traditional Tai Chi routine, the simplified Tai Chi exercise is more accessible to different skill levels of people (from novice to advanced level) and is more user-friendly, which is seemingly reflected by greater adherence (29 in the TCRT versus 23 in the TTC). When the traditional 85-form Tai Chi requires supervised practice because of the complex and lengthy routine, the simplified Tai Chi form can also be practiced at home because large space is not required. In addition, pushing hand in the simplified Tai Chi resistance training program provides practitioners with the opportunity to interact with each other, which makes the routine become more interesting and enjoyable.

## 5. Conclusions

Both the traditional and the simplified Tai Chi routines are effective in improving physical function in older adults, especially for physical balance. In the present study, the simplified Tai Chi outperforms the traditional Tai Chi. The simplified Tai Chi exercise may be an effective alternative method to strengthen physical function in older adults. Future studies should recruit a large number of participants with a mixed method to examine whether the effect of the simplified Tai Chi on health outcomes is superior to the traditional Tai Chi when comparing dropout rates and compliance and learnability.

## Figures and Tables

**Table 1:** Demographic information of participants at the baseline (mean ± SD).

Variables	TCRT, *n* = 30	TTC, *n* = 30	Control group, *n* = 30
Height (cm) Weight (kg) Age (years) BMI (kg/m^2^)	162.3 ± 4.9 62.3 ± 7.4 65.3 ± 4.3 23.6 ± 2.3	161.2 ± 6.1 59.6 ± 7.1 65.2 ± 5.0 22.9 ± 2.6	162.3 ± 5.5 63.3 ± 8.1 65.3 ± 4.4 23.7 ± 2.7

*Note.* SD: standard deviation; BMI: body mass index.

**Table 2:** The similarities and differences between simplified Tai Chi exercise (TCRT) and traditional Tai Chi (TTC).

Variables	Name of Tai Chi routine	
	TCRT	TTC
Origin	It was developed by a Qigong master at Shanghai Sport University in a recent year, simplified resistance Tai Chi	It was created by Chinese martial artists and has a history of more than 100 years, Traditional Yang Style 85-form Tai Chi.
Number of moves	32	85
Element of routine	Movements focus on weight shift, muscle stretching, cultivating internal energy, and fall-prevention training. In particular, pushing hands as a key element is added in which two practitioners place their dominant hands together and apply their force or action to make the opponent lose balance.	Hand movements are characterized by circulation, spiral, slowness, smoothness. low limbs are involved with weight-shift training while maintaining core stability.
Duration of routine	28 min, 30 seconds	28 min, 16 seconds
Exercise intensity	Moderate	Moderate
Strengths and Weaknesses	Relatively short routine is suitable for older adults due to age-related memory decline. Practitioners can carry out home-based practice, instructed by watching a video. Pushing hand provides practitioners with the opportunity to socialize with each other.	Lengthy routine is a challenge for older adults. Additionally, complex moves not only require supervised practice, but also could discourage novice practitioners (discontinue Tai Chi training).

**Table 3:** Changing conditions of the 7 FMS movement modes before and after the intervention.

Variables	TCRT			TTC			Control		
	Improve%	Keep%	Decline%	Improve%	Keep%	Decline%	Improve%	Keep%	Decline%
Deep squat	6.90	89.66	3.45	4.35	91.30	4.35	3.70	85.19	11.11
Hurdle step	37.93	58.62	3.45	26.09	69.57	4.35	14.81	81.48	3.70
In-line lunge	10.34	68.97	20.69	13.04	82.61	4.35	3.70	77.78	18.52
Shoulder mobility	24.14	68.97	6.90	52.17	43.48	4.35	18.52	66.67	14.81
Leg rise	10.34	86.21	3.45	8.70	82.61	8.70	18.52	62.96	18.52
Push-up	6.90	89.66	3.45	8.70	86.96	4.35	7.41	77.78	14.81
Rotary	13.79	86.21	0.00	13.04	82.61	4.35	18.52	77.78	3.70

## References

[1] Rubenstein L. Z. (2006). Falls in older people: epidemiology, risk factors and strategies for prevention. *Age and Ageing*.

[2] Studenski S., Duncan P. W., Chandler J. (1994). Predicting falls: the role of mobility and nonphysical factors. *Journal of the American Geriatrics Society*.

[3] Dite W., Temple V. A. (2002). A clinical test of stepping and change of direction to identify multiple falling older adults. *Archives of Physical Medicine and Rehabilitation*.

[4] Gillespie L. D., Robertson M. C., Gillespie W. J. (2009). Interventions for preventing falls in older people living in the community. *Cochrane Database of Systematic Reviews*.

[5] Li J. X., Hong Y., Chan K. M. (2001). Tai chi: physiological characteristics and beneficial effects on health. *British Journal of Sports Medicine*.

[6] Low S., Ang L. W., Goh K. S., Chew S. K. (2009). A systematic review of the effectiveness of Tai Chi on fall reduction among the elderly. *Archives of Gerontology and Geriatrics*.

[7] Schleicher M. M., Wedam L., Wu G. (2012). Review of Tai Chi as an effective exercise on falls prevention in elderly. *Research in Sports Medicine*.

[8] Li F., Harmer P., Fisher K. J., Mcauley E. (2004). Tai Chi: improving functional balance and predicting subsequent falls in older persons. *Medicine and Science in Sports & Exercise*.

[9] Wu G. (2002). Evaluation of the effectiveness of Tai Chi for improving balance and preventing falls in the older population—a review. *Journal of the American Geriatrics Society*.

[10] Minick K. I., Kiesel K. B., Burton L., Taylor A., Plisky P., Butler R. J. (2010). Interrater reliability of the functional movement screen. *The Journal of Strength and Conditioning Research*.

[11] Wadsworth C. T., Krishnan R., Sear M., Harrold J., Nielsen D. H. (1987). Intrarater reliability of manual muscle testing and hand-held dynametric muscle testing. *Physical Therapy*.

[12] Folstein M. F., Robins L. N., Helzer J. E. (1983). The mini-mental state examination. *Archives of General Psychiatry*.

[13] Teyhen D. S., Shaffer S. W., Lorenson C. L. (2012). The functional movement screen: A Reliability Study. *Journal of Orthopaedic and Sports Physical Therapy*.

[14] Day L., Fildes B., Gordon I., Fitzharris M., Flamer H., Lord S. (2002). Randomised factorial trial of falls prevention among older people living in their own homes. *British Medical Journal*.

[15] Barnett A., Smith B., Lord S. R., Williams M., Baumand A. (2003). Community-based group exercise improves balance and reduces falls in at-risk older people: a randomised controlled trial. *Age and Ageing*.

[16] Wang H., Yu B., Chen W., Lu Y., Yu D. (2015). Simplified Tai Chi resistance training versus traditional Tai Chi in slowing bone loss in postmenopausal women. *Evidence-Based Complementary and Alternative Medicine*.

[17] Sjösten N. M., Salonoja M., Piirtola M. (2007). A multifactorial fall prevention programme in home-dwelling elderly people: a randomized-controlled trial. *Public Health*.

[18] Shih J. (1997). Basic Beijing twenty-four forms of T'ai Chi exercise and average velocity of sway. *Perceptual and Motor Skills*.

[19] Forrest W. R. (1997). Anticipatory postural adjustment and T'ai Chi Ch'uan. *Biomedical Sciences Instrumentation*.

[20] Vaapio S., Salminen M., Vahlberg T. (2007). Effects of risk-based multifactorial fall prevention on health-related quality of life among the community-dwelling aged: a randomized controlled trial. *Health and Quality of Life Outcomes*.

